# Synthesis, Characterization, and Biological Evaluation of Noble Metal Complexes with 2‑(1-Benzyl‑1*H*‑1,2,3-triazol-4-yl)pyridine Ligand: An Interesting Class of Metallo-antimicrobial and -antitumor Agents

**DOI:** 10.1021/acsomega.6c00770

**Published:** 2026-04-16

**Authors:** Daniela Giunta, Maurizio Solinas, Bruna Canu, Maria I. Pilo, Alessandra Scano, Germano Orrù, Sara Fais, Giuseppina Pichiri, Antonio Zucca

**Affiliations:** † Institute of Biomolecular Chemistry, 9327 National Research Council (CNR), Trav. La Crucca 3, 07100, Sassari, Italy; ‡ Department of Chemical, Physical, Mathematical and Natural Sciences, 9312 University of Sassari, Via Vienna 2, 07100 Sassari, Italy; § Department of Surgical Sciences, 3111 University of Cagliari, 09124 Cagliari, Italy; ∥ Department of Medical Sciences and Public Health, University of Cagliari, 09124, Cagliari, Italy

## Abstract

The rise of antimicrobial resistance (AMR) and Multidrug Resistance (MDR) has compelled the scientific community to search for new families of antimicrobial agents. Coordination compounds of noble metals have been shown to be good candidates, not only as novel antimicrobials but also as antitumor drugs. Herein we report the synthesis of a series of palladium­(II), platinum­(II) and gold­(III) chelated complexes with a triazole-pyridine ligand, 2-(1-benzyl-1*H*-1,2,3-triazol-4-yl)­pyridine, **5-TzPy**. The complexes were isolated and characterized in solution by means of NMR spectroscopy, and their antibacterial, antifungal, and cytotoxic activities were tested. Among them, cationic gold compound **5**, [Au­(**5-TzPy**)­Cl_2_]­[BF_4_], exhibited the best overall antimicrobial and antibiofilm activity. It proved to be the most potent compound, showing the lowest MIC and MBC values, and the greatest efficacy against biofilm formation. These findings suggest that its ionic charge and chemical configuration enhance molecular diffusion and interaction with microbial cells. In addition, electrochemical data show a redox behavior for complex **5** which may be associated with an oxidative stress mechanism of action. Additionally, the study was extended to include preliminary assessments of antitumor activity. Cytotoxicity tests on human tumor (HT29) cells showed that complexes **3**, [Pt­(**5-TzPy**)­Me_2_], and **5**, [Au­(**5-TzPy**)­Cl_2_]­[BF_4_], exhibited the highest tumor cell toxicity. However, cytotoxicity studies on normal endothelial (EA.hy926) displayed a more favorable selectivity profile for complex **2**, [Pt­(**5-TzPy**)­Cl_2_]. Its moderate toxicity toward tumor cells and minimal effects on normal cells highlight the potential of **2** as a selective and safer antitumor agent.

## Graphical abstract

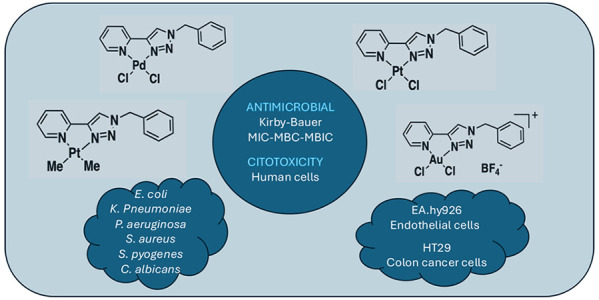

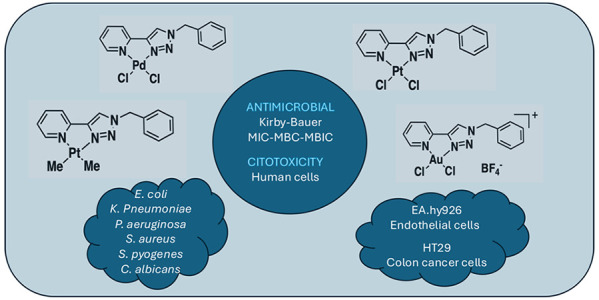

## Introduction

Coordination complexes of transition metals have become indispensable in various fields of science and technology. This class of compounds has, indeed, experienced an explosive growth over the past decades finding applications in advanced material science, industrial catalytic processes either homogeneous and heterogeneous, diagnostic imaging, energy storage and so on.^1^ Moreover, as a result of the growing demand for increasingly specific and selective drugs and the incredible progress made in understanding the mechanisms underlying diseases, a current field of application of metal complexes is medicinal chemistry. In this context, the possibility of tuning its reactivity toward electrophiles or nucleophiles, and to modify the three-dimensional geometries and redox capability by variations at either the metal center or the ligands, could be crucial to improve a potential therapeutic application of a transition metal complex.^2^ More in detail, opportune structural modification around the metal center could affect the pharmacokinetics and pharmacodynamics of a potential new drug, e.g. by increasing solubility, activity, bioavailability and stability.^3^

In addition to classical, inorganic, and coordination compounds, organometallic complexes have also found application: the first example was Arsphenamine, also known as compound 606. Discovered by serendipity in 1909 by Ehrlich and co-workers, it was used as an effective antimicrobial drug against syphilis.^4^ Another milestone in the field of coordination compounds in medicinal chemistry is, without any doubt, the discovery of the cis-diammino­dichloro­platinum­(II) (known as *cisplatin*) in the late 1960s.^5^ Since then, biomedical applications of metal containing drugs have been extended to other targets, looking at, *inter alia*, their antimicrobial and antiviral activity.^6^ This is particularly important as global public health has just had to deal with the recent widespread Covid situation, while experiencing a quiet epidemic due to antimicrobial-resistant (AMR) bacteria and even multidrug-resistant (MDR) strains.^7^ As conventional antibiotics progressively lose their efficacy, the development of novel antimicrobial agents has become not only urgent but also essential.

The World Health Organization (WHO) has classified AMR bacteria as a global health emergency, with profound implications in medical, veterinary, food, and economic fields. Despite the increasing demand for new antimicrobials, investment committed to their discovery and development remains insufficient. New compounds, with innovative chemical structures and novel mechanisms of action, are critically needed to combat MDR pathogens evolving resistance to existing antibiotic classes. Such innovation is essential for restoring the efficacy of antimicrobial therapies and ensuring the long-term sustainability of infection control strategies.^8^ In this respect, recent studies have shown that coordination compounds of noble metals display promising biological effects, both *in vitro* and *in vivo*, deserving attention as a new and innovative class of antimicrobial agents.^9^ The research for new metal-based antimicrobial agents looks at metal ions, nanoparticles and metal complexes, with the development of the so-called ‘metallo-antibiotics’ and ‘metallo-antimicrobials’.^10^ Although none of these species is currently in the clinical phase, they do represent a group of compounds having the potential to become fundamental in the fight against antibiotic-resistance.

Among the various families of coordination compounds, pyridine-derived complexes are particularly promising. Pyridines are considered as privileged structures in medicinal chemistry: ubiquitous in nature (DNA, alkaloids, vitamins, coenzymes, etc.) they have been often used in the design of drug candidates such as antitumor, antibacterial, antifungal, antiviral, analgesic, anti-inflammatory and antidiabetic.^11^ Moreover, a combination of pyridines’ properties with those of noble-metal centers may open the way to the buildup of particularly active species. With this in mind, we have directed our attention to investigate pyridine-derived molecules that can be used as chelating ligands. Prototypical ligand in this context is 2,2′-bipyridine (Figure 1), “the most widely used ligand” in coordination chemistry.^12^ From this, an ample series of derivatives may be easily obtained by structural modifications or introduction of appropriate substituents at the pyridine rings, therefore tuning the stereoelectronic properties of the entire molecule^13–17^ to give complexes with promising antimicrobial and antitumor properties.^18–23^ A further approach is to couple one pyridine ring with a different N-heterocycle, pursuing the idea of developing innovative families of N^∧^N chelating ligands.^24,25^ Among the nitrogen-based ligands, 1,2,3-triazoles gained attention in the past decade, because of their structural versatility and their potential diverse biological activities,^26^ comprising antimicrobial activity.^27^ Their complexes with transition metals have also been widely studied, emerging as promising antimicrobial and anticancer agents, with activity profiles strongly dependent on both the metal center and ligand architecture.

**Figure 1:**
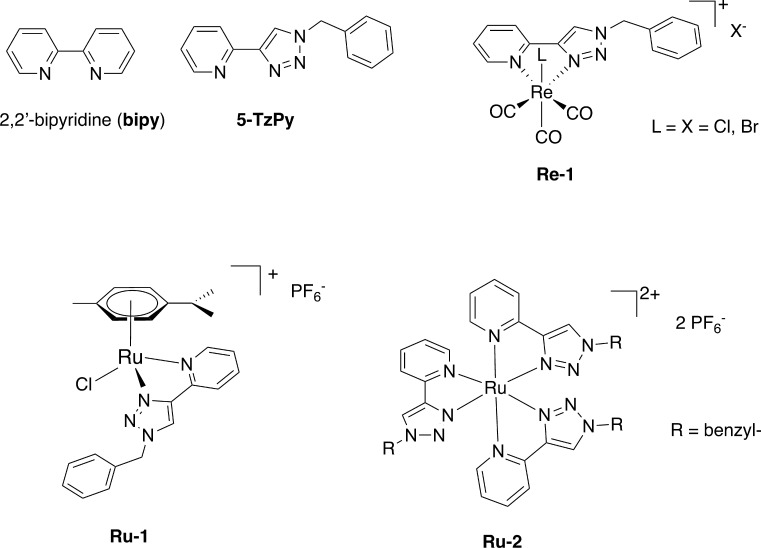
2,2′-Bipyridine (**bipy**) the prototypical N^∧^N chelating ligand; the 1,4-substituted-1,2,3 triazole-pyridine ligand (**5-TzPy**) studied in this paper; **Re-1**, **Ru-1** and **Ru-2**, representative Rhenium and Ruthenium complexes of 1,2,3-triazole-pyridine ligands with antimicrobial properties, investigated by Crowley,^29,44^ Webb^41^ and co-workers.

Transition metal complexes of N^∧^N bidentate chelators with 1,2,3-triazoles, especially pyridine-1,2,3-triazoles, have been reported to display various biological activities: among the various systems studied, several Ru­(II),^28,29,30^ Pd­(II),^31^ Mn­(I),^32^ Os­(II),^33^ Re­(I)^34^ complexes have shown significant antimicrobial effect toward Gram-positive and Gram-negative bacteria. Furthermore, very recently, a combinatorial approach allowed preparation of a vast library of bidentate pyridyl-1,2,3 triazole ligands. This ligand library was coordinated to five metal scaffolds to yield 672 metal compounds. Six promising metalloantibiotics of Re, Ir and Mn were identified and studied, exhibiting activity against Gram-positive bacteria in the nanomolar range.^35^

In addition, several complexes, including N^∧^N bidentate Rh­(III), Ir­(III), Ru­(II), and Os­(II), as well as monometallic Re­(I) and heterobimetallic Re­(I)/Fe­(II) complexes with 1,2,3-triazoles displayed significant anticancer effects.^36,37^ So far, the *in vitro* cytotoxicity of Pt­(II) complexes with bidentate pyridyl-1,2,3-triazole ligands, including the ligand herein studied, **5-TzPy**, was tested against a series of cell lines, showing good efficacy against HT29 colon carcinoma and Du145 prostate cancer.^35^

Some of the authors have found that cationic complexes exhibit superior activity compared to neutral analogs.^34^ Is is also worth noting that some of the reported compounds are also active against Gram-negative bacteria, which are generally more resistant to treatments.^32^

## Mechanistic Connections Between Antimicrobial and Anticancer Activity^34^

A compelling feature of transition metal complexes and, in particular, of triazole derivatives is their frequent exhibition of dual antimicrobial and anticancer activity, a phenomenon that reflects overlapping molecular targets and mechanisms of action in bacterial and cancer cells. This dual bioactivity is not coincidental but arises from fundamental similarities in the cellular vulnerabilities exploited by these complexes. These mechanistic connections provide a rational basis for the parallel development of metal-based therapeutics for infectious diseases and oncology.

DNA represents a critical target for both antimicrobial and anticancer metal complexes. As an example, Copper­(II) triazolopyrimidine complexes synthesized by Ruta and colleagues exhibited DNA intercalating capacity and nuclease-like activity, contributing to both their antiproliferative effects and antimicrobial activity.^18^ The complexes showed selective toxicity, suggesting that rapidly dividing cells—whether bacterial or cancerous—are preferentially targeted due to their heightened DNA replication and repair demands.

Other major mechanisms leading to antimicrobial and anticancer activity comprise membrane targeting, redox activity and generation of reactive oxygen species (ROS) that damage multiple cellular targets.^18,39^

In the present manuscript, we report, as outcomes of a multidisciplinary project, the synthesis and biological activity of noble metal complexes containing a 1,4-substituted-1,2,3-triazole-pyridine ligand. More in detail, we describe the results obtained with the 2-(1-benzyl-1*H*-1,2,3-triazol-4-yl)­pyridine, **5-TzPy** (Figure 1), a N^∧^N chelating ligand whose transition metal complexes have recently demonstrated potent antimicrobial activity.^29,33,36,40,41^ Representive structures of antimicrobial pyridine-triazole complexes are reported in Figure 1, illustrating the diversity of coordination modes and metal centers employed in recent studies.

As reported above, studies have revealed that transition metal complexes with nitrogen ligands possess the capacity to exhibit both antimicrobial and anticancer activity.^22^ Notably, compounds initially designed for one purpose (e.g., as antimicrobials) may demonstrate additional properties, such as anticancer activity, upon re-evaluation in alternative biological contexts.^42^ Inspired by the concept of reusing existing chemical structures as an efficient strategy to accelerate drug discovery and development,^43^ we decided to explore not only the potential antimicrobial activity of the synthesized metal complexes but also their potential as anticancer agents against colon cancer cells.

## Results and Discussion

### Chemistry

The 2-(1,2,3-triazol-4-yl)­pyridine family of ligands has become an important class of chelating ligands widely used for the synthesis of coordination and supramolecular complexes with interesting catalytic, biological, magnetic, electrochemical and photophysical properties.^45^ In this context, the 2-(1-benzyl-1*H*-1,2,3-triazol-4-yl)­pyridine (**5-TzPy**) ligand, reported here, has been scarcely studied with noble metals such as iridium,^46^ copper,^47^ and in particular platinum, palladium and gold.^48,49^ So far, the *in vitro* cytotoxicity of Pt­(II) complexes with bidentate pyridyl-1,2,3-triazole ligands, including **5-TzPy**, was tested against a series of cell lines, showing good efficacy against HT29 colon carcinoma and Du145 prostate cancer.^38^

Crowley and co-workers have recently reported the synthesis and the initial results obtained with the study of the antimicrobial properties of ruthenium pyridyl-triazole complexes, particularly against pathogenic bacteria, including *Staphylococcus aureus*,^40^ as well as some interesting results with platinum complexes with cancer cell lines.^32^

Starting from this background, in line with the necessity of more sustainable approach to drugs synthesis, **5-TzPy** was here synthesized by a modification of the reported procedure, which involves the copper catalyzed azide alkyne cycloaddition (CuAAC), “click reaction” (Scheme 1).^50,51^ This straightforward synthetic route enables the preparation of pyridine-triazole ligands, which may be studied as readily functionalized 2,2’-bipyridine analogues. However, differently from what was previously described by Crowley and co-workers, we obtained the ligand in a pure form through a *one-pot two-step* reaction performed in a 1/1 mixture of *tert*-butanol and water. The procedure starts from the conversion of the benzyl bromide into the corresponding azide. After addition of a stoichiometric amount of AgNO_3_, followed by proper filtration of the resulting AgBr salt,^52^ the benzyl azide can react with the 2-ethynylpyridine under Sharpless’s reaction conditions (Cu­(OAc)_2_·H_2_O, NaAsc, ^*t*^BuOH/H_2_O). This synthetic strategy allows us to isolate pure product by a simple aqueous/organic workup, avoiding expensive and time-consuming purification steps, such as flash chromatography.

**Scheme 1:**
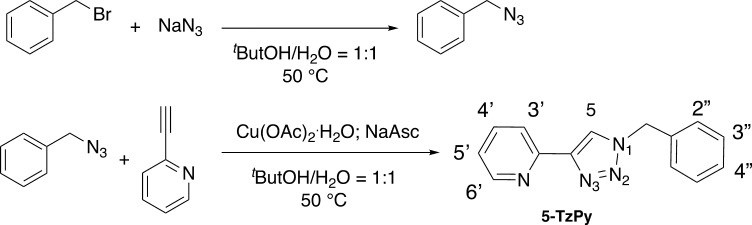
Synthesis of 2-(1-Benzyl-1*H*-1,2,3-triazol-4-yl)­pyridine (**5-TzPy**) with Numerical Scheme for NMR Discussion (NaAsc = Sodium Ascorbate)

The **5-TzPy** chloride complexes, [Pd­(**5-TzPy**)­Cl_2_] (**1**) and [Pt­(**5-TzPy**)­Cl_2_] (**2**) (Figure 2), were first synthesized by Sarkar and co-workers in 2009 ^48^ starting from [Pd­(COD)­Cl_2_] and [Pt­(DMSO)_2_Cl_2_], respectively (COD = 1,5-cyclooctadiene, DMSO = dimethyl sulfoxide). They reported on the resolution of the X-ray crystal structures of both complexes, therefore highlighting a greater *trans*-influence of the triazole nitrogen N^3^ as compared with that observed for nitrogen at the pyridine ring. This effect resulted in a different length of the M–Cl bonds. Moreover, the X-ray structures revealed an elongation of the N^3^–N^2^ bonds because of the back-donation from the metal. Successively, complexes **1** and **2** were also synthesized by Kilpin and Crowley in 2010,^49^ following different synthetic methods, i.e. starting from [Pd­(CH_3_CN)_2_Cl_2_] and K_2_PtCl_4_. Interestingly, no antimicrobial activity was investigated for **1** and **2** and to the best of our knowledge, the study on the biological activity of these complexes is limited to a test on the cytotoxicity of **5-TzPy** and **1**: in contrast to a series of related palladium complexes bearing a carbohydrate scaffold, they reveal no inhibitory activity on cancer lines.^53^ Thus, to investigate the antimicrobial properties of chelated **5-TzPy** complexes with d^8^ transition noble metals, we prepared complexes **1** and **2** following the Sarkar methods, simply changing the solvent for the second (**2**, acetone solvent in place of refluxing nitromethane).

**Figure 2:**
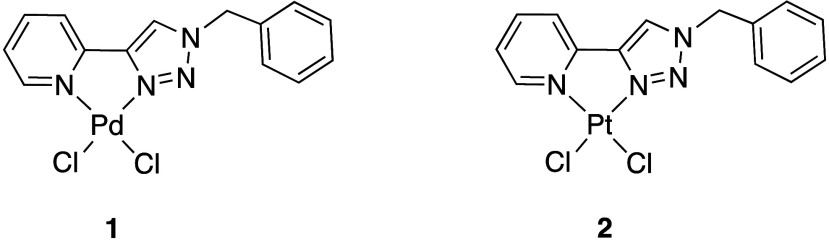
Complexes **1** and **2**.

Analysis of the ^1^H NMR spectra of **1** and **2** showed the expected signals, in agreement with published data. A noteworthy feature, revealed in the spectra, is the coordination shift of the H^5^ proton of the triazole ring: this signal, appearing as a singlet, is strongly deshielded after coordination, resonating at 9.21 and 9.24 ppm in complexes **1** and **2**, respectively (Δδ = 0.77 and 0.80 ppm compared to the simple ligand). This strong coordination shift may arise from various factors, all indicating chelation.

Palladium and platinum are often described as similar metals. However, platinum displays a more complex behavior due to its higher electronegativity and relativistic effects, as disclosed by inverse-crystal field theory. This particular feature is shared with gold.^54–57^ Differently from palladium­(II), platinum­(II) may, indeed, show both nucleophilic or electrophilic character, depending on the ligand’s properties. For this reason, [Pt­(N^∧^N)­Cl_2_] and [Pt­(N^∧^N)­Me_2_] complexes typically show distinct and contrasting behaviors.^58^ With the intention to compare the electron-poor complex **2** with an electron-rich analogue, we reacted [Pt­(DMSO)_2_Me_2_] with the **5-TzPy** ligand in acetone, yielding [Pt­(**5-TzPy**)­Me_2_]­(**3**) in good yields (Scheme 2).

**Scheme 2:**
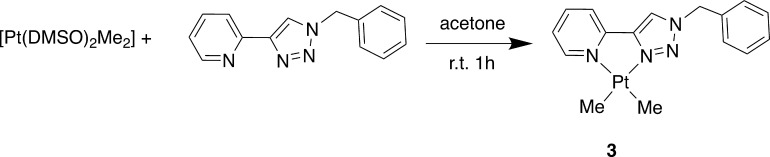
Synthesis of Complex **3**

Complex **3** has been thoroughly characterized by means of 1D and 2D NMR spectroscopy. The ^1^H NMR spectrum shows the expected 10 aromatic signals and benzylic CH_2_ for the coordinated **5-TzPy** ligand: the H^6′^ proton is strongly deshielded, appearing as a multiplet with satellites at *δ* = 9.12 ppm. The small value of the Pt–H coupling (^3^J_Pt–H_ = 20 Hz), measured through these satellites, demonstrates coordination of the pyridine nitrogen in *trans* to a donor with a great *trans* influence, such as a methyl group. The coordinated methyls appear as singlets with satellites at 1.34 (^2^J_Pt–H_ = 91.0 Hz) and 1.05 (^2^J_Pt–H_ = 88.3 Hz) ppm, respectively. Here the Pt–H coupling constants are in agreement with a coordination in *trans* to nitrogen atoms with different *trans* influence as previously revealed by X-ray analysis.^48^ For complex **3**, the H^5^ proton signal is not particularly deshielded (δ = 7.81 ppm), and this appears anomalous in comparison to that observed for **2** ([Pt­(**5-TzPy**)­Cl_2_], δ= 9.24 ppm). However, this different behavior may be related to the electron-rich nature of the platinum center, which may be involved in a strong back-donation to the chelating ligand. To complete the characterization, complex **3** was subjected to two-dimensional H–H COSY and NOESY experiments. The COSY spectrum allowed assignment of ^1^H resonances, and the NOESY spectrum showed all the expected through-space correlations (see Figure 3), such as the correlation between the Pt-CH_3_ at 1.05 ppm and the H^6′^ proton at 9.24 ppm. From these, it appears that the nitrogen in *trans* to the methyl, i.e. the one with the highest *trans* influence, is the triazole N^3^. This agrees with the relatively small ^2^J_Pt–H_ value of 88.3 Hz. The NOESY spectrum also shows a weak contact between the H^3′^ and H^5^ singlets, thus confirming chelation (see Figure 3).

**Figure 3:**
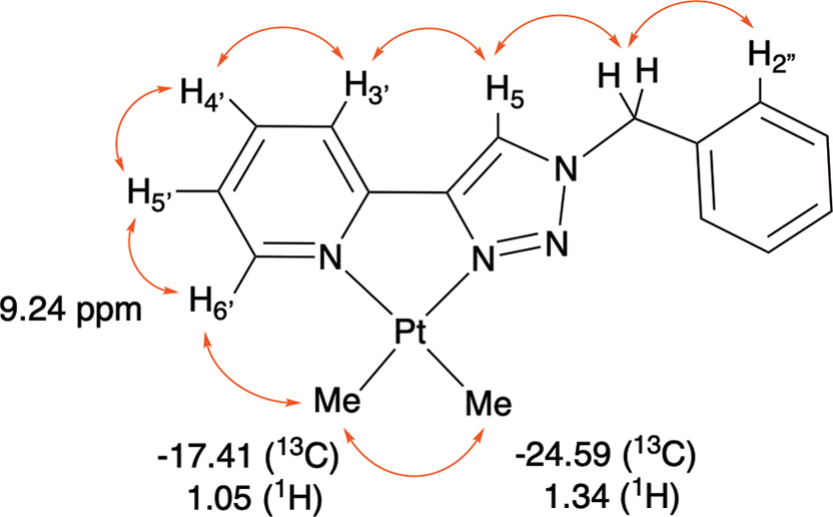
Complex **3**, [Pt­(**5-PyTz**)­Me_2_]: the red arrows represent the Noe contacts revealed in the NOESY spectrum. ^1^H–^13^C correlations of methyl groups are also reported below.

The H–C heterocorrelated HMBC and HSQC spectra allowed attribution of all ^1^H and ^13^C NMR signals. In particular, the HSQC spectrum showed direct correlation of the CH_3_ hydrogens with the corresponding carbon (δ −17.41 ppm, 1.05 ppm; −24.59 ppm, 1.34 ppm). Accordingly, the long-distance heterocorrelated experiment, HMBC, presents the crossed pairings of the hydrogens of each methyl with the carbon of the other methyl, i.e., δ −17.41 ppm (^13^C) with 1.34 ppm (^1^H) and −24.59 ppm (^13^C) with 1.05 ppm (^1^H). The one-dimensional ^13^C NMR spectrum, despite long acquisition times, did not allow resolution of satellites and determination of ^195^Pt–^13^C couplings. However, approximate data were obtained from the 2D HSQC spectrum: CH_3_ *trans* to N^3^ δ = 17.41, ^1^J_Pt–C_ = 860 Hz, CH_3_ *trans* to N­(Py) δ = 24.59 ppm, ^1^J_Pt–C_ = 760 Hz.

The investigation was extended to a gold­(III) complex: the reaction of Na­[AuCl_4_] and **5-TzPy** in a H_2_O/CH_3_CN 3/1 mixture, at room temperature, gave the monodentate adduct [Au­(**5-TzPy**)­Cl_3_], **4** (Scheme 3), although not in pure form. The ^1^H NMR spectrum of **4** shows, indeed, all the expected signals for the given formulation, displaying, *inter alia*, a downfield shift of the H^6′^ signal (δ = 9.22 ppm) arose from coordination, as previously reported for analogous monodentate Au­(III)-pyridine adducts.^22^ Overall, here the spectrum reveals the presence of a second species (5% ca.) likely corresponding to the chelated adduct [Au­(N^∧^N)­Cl_2_]^+^: this seems to indicate a good propensity of the **5-TzPy** ligand to chelate. In line with this, an acetone solution of complex **4** displays low conductivity, reflecting the presence of small amounts of the cationic adduct. A complete conversion of the monodentate adduct to the chelate complex was achieved by further reaction of **4** with AgBF_4_. As shown in Scheme 3, after abstraction of one chloride ligand, the desired cationic chelated complex [Au­(**5-TzPy**)­Cl_2_]­BF_4_ (**5**) was then isolated as a yellow solid. The ^1^H NMR spectrum of **5** shows all the expected signals, with two strongly deshielded signals, corresponding to the H^5^ (δ 9.57 ppm, *s*) and H^6′^ (9.51 ppm, *dd*) protons. ^1^H NMR analysis confirms that the N^∧^N chelated complex **5** was present in small quantities already in the synthesis of **4**. Moreover, a comparison between the Au­(III) complexes’ ^1^H NMR signals reveals how, after chelation, the singlet for H^5′^ moves to higher chemical shifts: δ = 8.12 ppm for simple **5-TzPy** moves to 9.06 for complex **4** and 9.57 ppm for **5**.

**Scheme 3:**
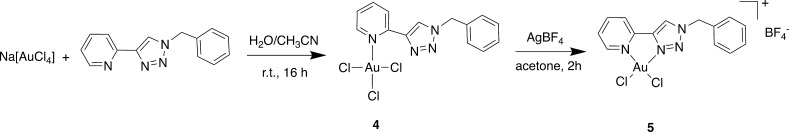
Synthesis of Complexes **4** and **5**

Recently, Griffith and co-workers reported the synthesis of a gold­(III) complex with 2-(1-phenyl-1*H*-1,2,3-triazol-4-yl)­pyridine,^59^ where the ligand exhibited a monodentate coordination mode, [Au­(N)­Cl_3_]. In our case, however, the use of AgBF_4_ was crucial in extruding a chloride ligand, forcing chelation and leading to the cationic [Au­(N^∧^N)­Cl_2_]^+^ species. Conductivity measurement in acetone, with a molar conductivity value typical of a 1:1 electrolyte,^60^ confirmed the electrolytic nature of complex **5**, supporting the proposed cationic structure.

As described below, complex **5** showed the best antimicrobial and antibiofilm activity. This complex belongs to a series of chelated gold­(III) complexes of general formula [Au­(N^∧^N)­Cl_2_]^+^, whose prototypical complexes are the 2,2’-bipyridine derivatives [Au­(bipy)­Cl_2_]^+^,^61^ which have showed important catalytic,^62^ antimicrobial^20^ and antitumor properties.^63^ For this reason, this complex was studied in detail also by means of cyclic voltammetry, in order to find insights into its chemical behavior.

### Electrochemistry

The voltammetric behavior of the Pd­(II) and Pt­(II) derivatives (**1** and **2**, respectively) was previously described.^48^ Both **1** and **2** show a single reduction process at highly negative potentials (−1.28 V and −2.15 V, respectively) in dichloromethane. On the other hand, to the best of our knowledge the electrochemical characterization of the analogue Au­(III) complex has not been yet reported. Therefore, here the voltammetric behavior of the Au­(III) complex **5** at a platinum working electrode has been investigated. In order to avoid coordination effects by the solvent system, dichloromethane was selected as the solvent in the electrochemical characterization. The cyclic voltammetric response at 100 mV s^–1^ as the potential scan rate shows a cathodic process at 0.06 V vs the ferrocene/ferricinium ion redox couple, with an associated process in the reverse anodic scan at 0.64 V (Figure 4 – inset). A further quasi-reversible process occurs at −0.26 V with a reverse peak at −0.12 V, and the presence of a thin gold-film is observed on the electrode surface. Two additional cathodic processes are observed, the first one at −0.48 V with a quasi-reversible behavior (reverse peak at −0.34 V), and the last (irreversible) one at −0.92 V. By reversing the potential scan direction up to 1 V, the anodic peak detectable at 0.64 V as associated with the first reduction process splits into two peaks at 0.52 and 0.77 V, respectively, with the first one slightly sharper than the second one when the scan is reversed after the second cathodic peak (0.26 V). On the other hand, when the scan is reversed after the last two cathodic peaks (−0.48 V and −0.92 V) the anodic peak at 0.52 V becomes increasingly sharper. The analysis of the voltammetric pattern suggests ascribing the less cathodic peaks (0.06 and 0.64 V) to a two-step process where a Au­(III)→Au­(I) reduction is followed by a Au­(I)→Au(0) step, with an oxidative desorption process in the reverse anodic scan evidenced by a typical sharp peak. The proposed Au­(III)→Au­(I)/Au­(I)→Au(0) reduction process is supported by the comparison with N^∧^N Au­(III)-complexes previously reported.^61,64,65^ Cyclic voltammetry experiments carried out at different potential scan rates between 0.02 and 0.50 mV s^–1^ (Figure 4) evidence a linear relationship between the peak current of the less cathodic process and the scan rate square root, suggesting a diffusive feature of the first reductive electron transfer. As for the further more cathodic processes, they can be tentatively ascribed to rearrangements of the reduced Au(0) complex, causing the even sharper profile of the associated reverse peak. On the contrary, the more cathodic processes cannot be reasonably ascribed to the ligand reduction which usually happens at highly negative values^48,66^ not accessible in the solvent system here adopted.

**Figure 4:**
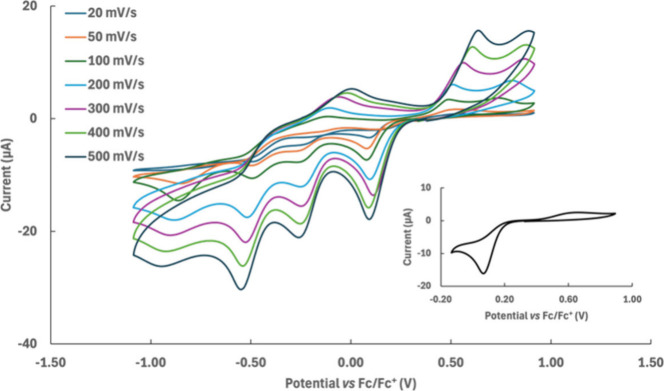
Cyclic voltammetry curves of complex **5** at potential scan rates between 20 mV s^–1^ and 500 mV s^–1^ in 0.1 M TEAPF_6_/CH_2_Cl_2_. Inset: cyclic voltammetry curve between −0.20 and 1.00 V; potential scan rate: 100 mV s^–1^.

### Biological Activity

The biological activity of transition metal complexes is routinely assessed using DMSO as a solvent. While this offers advantages such as facile dilution with aqueous media, DMSO can act as a competing ligand, potentially inducing ligand substitution reactions. This is a well-known phenomenon. In some cases, the active species in solution is not the same molecular entity isolated in the solid state; this is a common problem in catalysis and in biological studies. As an example, in the case of cisplatin, the active species differs from the form initially dissolved.^67,68^ Consequently, the stability of complexes **1**, **2**, **3**, and **5** in DMSO was examined by ^1^H NMR spectroscopy.

Complexes **1**, **2** and **5** ([M­(N^∧^N)­Cl_2_]) proved stable in DMSO, whereas complex **3** ([Pt­(N^∧^N)­Me_2_]) underwent DMSO-**5PyTz** ligand exchange regenerating the precursor [Pt­(DMSO)_2_Me_2_], according to the reaction [Pt­(**5-PyTz**)­Me_2_] + 2 DMSO ⇄ [Pt­(DMSO)_2_Me_2_] + **5-PyTz**.

This behavior is attributed to the equilibrium in the reaction that can be shifted in either direction, depending on the experimental conditions.

Dilution of a DMSO-d6 solution of **3** with D_2_O (1:10 DMSO-d6/D_2_O) revealed a dynamic behavior: a broad signal for the H^6^ proton at δ ∼ 8.8 ppm (δ 8.91 in **3**, δ 8.58 in the free ligand) indicative of probable complexation and dynamic behavior on the NMR time scale. Accordingly, biological testing included both solutions of complex **3**, free **5-TzPy** and [Pt­(DMSO)_2_Me_2_].

The development of the above-mentioned synthetic strategies opened the way to the study of the antimicrobial activity of complexes **1**–**3** and **5**, carried out in accordance with the procedures described by the Clinical and Laboratory Standards Institute (CLSI). Gram-positive bacteria (*Staphylococcus aureus* and *Streptococcus pyogenes*), Gram-negative bacteria (*Escherichia coli*, *Pseudomonas aeruginosa*, and *Klebsiella pneumoniae*), and the yeast *Candida albicans* were selected to assess whether the compounds exhibit broad-spectrum activity, selectivity toward specific pathogen classes, or properties making them promising lead compounds. This diverse panel provides a comprehensive preliminary evaluation of antimicrobial efficacy and insight into the potential therapeutic scope of the compounds. A first line of evaluation was performed with the agar diffusion test (Kirby–Bauer), as this procedure allows a rapid assessment of bacterial resistance or susceptibility to the compounds. Based on the Kirby–Bauer test, the ligand **5-TzPy** and compounds **1**, **2** and **3** did not show any activity against bacteria and yeast (inhibition diameter ∼0 mm). On the contrary, using the same method the gold complex **5** was revealed to be active against the Gram-positive (*S. aureus* and *S. pyogenes*) as well as the Gram-negative bacteria (*E. coli*, *P. aeruginosa* and *K. pneumoniae*) and *C. albicans* (Table 1), with the *S. pyogenes* being the most sensitive. In addition, the precursor [Pt­(DMSO)_2_(Me)_2_] was analyzed for its antimicrobial properties. The complex produced visible inhibition alone for *S. aureus* and *S. pyogenes* as shown in Table 1. Similar to the other platinum-based complex (**3**), [Pt­(DMSO)_2_(Me)_2_] failed to inhibit *P. aeruginosa*, *K. pneumoniae*, and *C. albicans* on agar likely due to steric hindrance or diffusion constraints within the agar matrix.

**Table 1:** Antimicrobial Profile of [Au­(**5-TzPy**)­Cl_2_]­BF_4_ (**5**) and [Pt­(DMSO)_2_(Me)_2_] (**3**) (c = 0.01 M) Performed with the Kirby–Bauer Test

		inhibition diameter, *mm*	
Strain		[Au(5-TzPy)Cl_2_]BF_4_	[Pt(DMSO)_2_(Me)_2_]
**Gram negative**	*E. coli*	18	0
	*P. aeruginosa*	20	0
	*K. pneumoniae*	20	0
**Gram positive**	*S. aureus*	17	12
	*S. pyogenes*	25	15
**Yeast**	*C. albicans*	15	0

Successively, a standard broth dilution method was used to study the antimicrobial efficacy of the compounds by evaluating the visible growth of microorganisms in nutrient broth.

Following these initial results, we further investigated the behavior of the selected pathogenic strains in the presence of the new complexes measuring their MIC (minimum inhibitory concentration), MBC and MBIC. In detail, initial evaluation revealed the lowest concentration of the tested molecules was able to inhibit visible growth of a fixed concentration of each strain (MIC). Tables 2 and 3 highlight that the lowest values were measured for *C. albicans*. Generally, after diluting the MIC broth, it is then determined whether the culture shows growth or not, thus measuring the lowest concentration of a potential antimicrobial able to kill 99.9% of the bacterial population (MBC: minimum bactericidal concentration): if, after dilution, the culture shows no growth, the bacterium has been killed, meaning the molecule is bactericidal, on the contrary is only bacteriostatic. Finally, the minimum inhibitory concentration of biofilm (MBIC) was also evaluated, with some modifications of the crystal violet staining protocol (http://www.biofilm.montana.edu). The MBIC represents the lowest concentration showing an absorbance comparable with the negative control (measured in absence of bacteria), evaluated in liquid medium (Tables 2 and 3).

**Table 2:** MIC, MBC and MBIC Values (mol/L) Measured for Complexes **1** and **2**

	1			2		
Compound	MIC	MBC	MBIC	MIC	MBC	MBIC
*E. coli*	5 × 10^–3^	5 × 10^–3^	>0.01	5 × 10^–3^	5 × 10^–3^	>0.01
*K. pneumoniae*	5 × 10^–3^	5 × 10^–3^	5 × 10^–3^	5 × 10^–3^	5 × 10^–3^	5 × 10^–3^
*P. aeruginosa*	>0.01	>0.01	>0.01	>0.01	>0.01	>0.01
*S. aureus*	2.5 × 10^–3^	2.5 × 10^–3^	>0.01	5 × 10^–3^	5 × 10^–3^	5 × 10^–3^
*S. pyogenes*	2.5 × 10^–3^	2.5 × 10^–3^	2.5 × 10^–3^	5 × 10^–3^	5 × 10^–3^	5 × 10^–3^
*C. albicans*	3.1 × 10^–4^	3.1 × 10^–4^	>0.01	1.25 × 10^–3^	1.25 × 10^–3^	>0.01

**Table 3:** MIC, MBC and MBIC Values (mol/L) Measured for Complexes **3** ^a^ and **5**

	3			5		
Compound	MIC	MBC	MBIC	MIC	MBC	MBIC
*E. coli*	2.5 × 10^–3^	2.5 × 10^–3^	1.25 × 10^–3^	>0.01	>0.01	>0.01
*K. pneumoniae*	5 × 10^–3^	5 × 10^–3^	5 × 10^–3^	6.2 × 10^–4^	6.2 × 10^–4^	6.2 × 10^–4^
*P. aeruginosa*	>0.01	>0.01	>0.01	>0.01	>0.01	>0.01
*S. aureus*	2.5 × 10^–3^	2.5 × 10^–3^	5 × 10^–3^	3.1 × 10^–4^	3.1 × 10^–4^	6.2 × 10^–4^
*S. pyogenes*	2.5 × 10^–3^	2.5 × 10^–3^	>0.01	6.2 × 10^–4^	6.2 × 10^–4^	1.25 × 10^–3^
*C. albicans*	1.5 × 10^–4^	1.5 × 10^–4^	>0.01	1.5 × 10^–4^	1.5 × 10^–4^	>0.01

a As stated above, solutions of complex **3** in DMSO/water likely contain [Pt­(DMSO)_2_Me_2_] and dynamic complexes of Pt and **5-PyTz**.

As reported in Tables 2 and 3 these compounds caused bacterial growth inhibition (MIC) in a concentration range from 5 × 10^–3^ M to 1.5 × 10^–4^ M. Thus, all the tested molecules show some bactericide activity, with *C. albicans* representing the most sensitive strain. These results also highlight a significant difference between the four complexes in the ability to interfere in the biofilm formation (MBIC). As shown in Table 3, complex **5** gave lower MBIC values (6.2 × 10^–4^) than **1** (2.5 × 10^–3^), **2** (5 × 10^–3^) and solutions of **3** (1.25 × 10^–3^). For *E. coli* and *C. albicans*, MBIC values are higher compared to MIC/MBC, suggesting that, for these strains, the biofilm state confers enhanced resistance to the compounds. In contrast, *K. pneumoniae*, *S. aureus*, and *S. pyogenes* showed similar MBICs and MIC values, implying that the metal complexes can effectively prevent biofilm formation in these organisms.

Thus, differently from the Kirby–Bauer test, antimicrobial and antibiofilm analyses bring to light appreciable activities also for compounds **1**, **2** and **3** (Tables 1–3). Possibly a steric hindrance in the agar’s structure may prevent diffusion of the tested molecules. We do guess that a possible explanation of this different behavior may be related to the ionic nature of complex **5**, compared to **1**, **2** and **3**.

Overall, the data suggest that either the chemical structure and the physicochemical properties of the tested molecules plays a critical role in their effectiveness against planktonic and biofilm-embedded microbial populations. In this context, *Pseudomonas aeruginosa* deserves attention, as it showed an inhibition halo in the agar diffusion test, but no measurable MIC, MBC, or MBIC values (>0.01 M). In the Kirby–Bauer test, the compound can locally inhibit bacterial growth where its surface concentration is highest, resulting in a visible inhibition zone. However, in the broth dilution method, *P. aeruginosa* shows intrinsic resistance due to its low outer membrane permeability, active efflux systems, and strong biofilm formation. These features limit the penetration of the compound and significantly increase the tolerance. Therefore, apparent inhibition on solid media does not necessarily translate into actual antimicrobial efficacy under liquid culture or biofilm conditions.^69^

An interesting result was obtained by antimicrobial dilution tests of the precursor [Pt­(DMSO)_2_Me_2_]. The complex inhibited *P. aeruginosa* with a MIC of 1.25 × 10^–3^ M (Table 4), a strain that was completely resistant to all other gold and platinum complexes investigated in this study (all >0.01 M). *S. pyogenes* proved to be extremely sensitive to the Pt­(DMSO)_2_Me_2_ complex, exhibiting a Minimum Bactericidal Concentration (MBC) of 7.81 × 10^–5^ M, which is significantly more potent than that observed for solutions of complex **3** (2.5 × 10^–3^ M). For *E. coli*, [Pt­(DMSO)_2_Me_2_] (6.25 × 10^–4^ M) was approximately four times more effective than complex **3**. The behavior of [Pt­(DMSO)_2_Me_2_] in biofilm assays highlights a trade-off between the bacterial and fungal efficacy. For all bacterial strains, the MBIC was >0.01 M, suggesting that while [Pt­(DMSO)_2_Me_2_] is highly effective against planktonic bacteria, it is unable to effectively penetrate or inhibit the bacterial biofilm matrix. Remarkably, [Pt­(DMSO)_2_Me_2_] was the only complex in the series (including **1**, **2**, **3**, and **5**) to show activity against the biofilm of *C. albicans*, with an MBIC of 6.25 × 10^–4^ M. This finding suggests a unique mechanism of action against fungal extracellular matrices that is not shared by the other complexes. The data suggest that the DMSO ligands in [Pt­(DMSO)_2_Me_2_] play a critical role in broadening the antimicrobial spectrum to include *P. aerug*inosa and providing a rare efficacy against *Candida* biofilms. However, its lack of activity against bacterial biofilms indicates that the chemical structure of the metal complex (gold vs. platinum) and the nature of the ligands (ionic vs neutral) remain the deciding factors in therapeutic scope.

**Table 4:** MIC, MBC and MBIC Values (mol/L) Measured for [Pt­(DMSO)_2_Me_2_]

	Pt(DMSO)_2_(Me)_2_		
Compound	MIC	MBC	MBIC
*E. coli*	6.25 × 10^–4^	1.25 × 10^–3^	>0.01
*K. pneumoniae*	1.25 × 10^–3^	2.5 × 10^–3^	>0.01
*P. aeruginosa*	1.25 × 10^–3^	2.5 × 10^–3^	>0.01
*S. aureus*	2.5 × 10^–3^	2.5 × 10^–3^	>0.01
*S. pyogenes*	3.13 × 10^–4^	7.81 × 10^–5^	>0.01
*C. albicans*	6.25 × 10^–4^	6.25 × 10^–4^	6.25 × 10^–4^

This result appears very interesting. *cis*-[Pt­(DMSO)_2_Me_2_] is a common metal precursor for organometallic complexes, but to the best of our knowledge, its antimicrobial activity has never been reported. These data open the way for future investigations on the family of electron-rich *cis*-[Pt­(DMSO)_2_R_2_] (R = Me, Ph, etc) complexes, in some way related to the highly studied, electron-poor cisplatin, *cis*-[Pt­(NH_3_)_2_Cl_2_]. A relationship between the biological behavior in solution of complex **3** and its precursor [Pt­(DMSO)_2_Me_2_] appears complex and deserves future investigation.

The toxicity assay was used to determine the potential of these new chemicals to be hazardous to human cells. We were using standard protocols, based on tissue cells *in vitro*, to study morphological effects of cells growth induced by these compounds. The *in vitro* colorimetric MTT-assay (MTT = 3-(4,5-dimethyl-2-thiazolyl)- 2,5-diphenyl-2H-tetrazolium bromide) used here allows a rapid evaluation of the cytotoxicity of the complexes and their effect on cell proliferation. It is particularly useful when evaluating molecular abilities related to cell survival and/or growth inhibition. We initially used normal endothelial EA.hy926 cell lines to study the cytotoxicity of complexes **1**, **2**, **3** and **5**. At the same time, their behavior with the HT29 colon cancer cell lines gives an idea of any potential antitumor activity. Therefore, increasing amounts (1, 3, 5, and 7 μL) of complexes **1**, **2**, **3** and **5** (C = 0.01 M) were added to both cell lines, and after 24 h, the MTT viability test was performed using the cell proliferation Kit I (MTT). The viability control value obtained in untreated cells was considered as 100% of viability. The results are shown in Figures 5 and 6.

**Figure 5:**
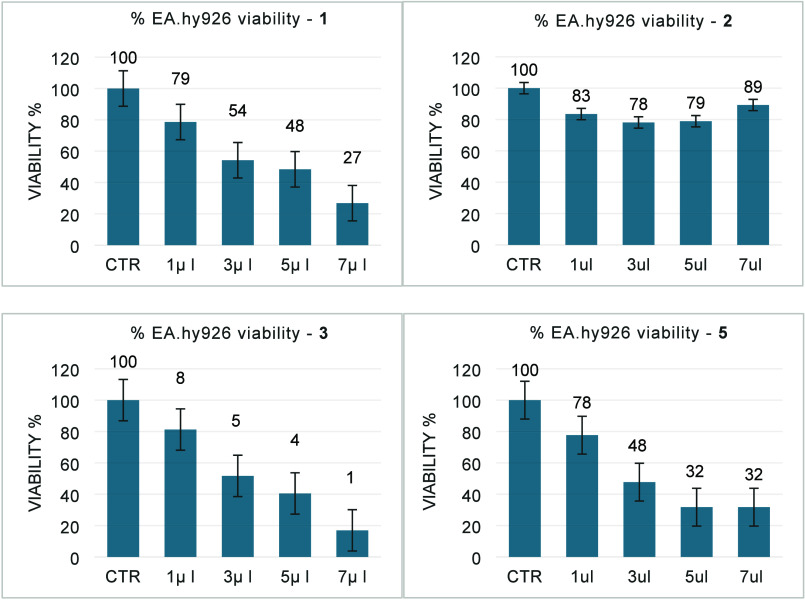
Effect of **1** [Pd­(**5-TzPy**)­Cl_2_], **2** [Pt­(5-Tzpy)­Cl_2_], **3** [Pt­(**5-TzPy**)­Me_2_] and **5** [Au­(**5-TzPy**)­Cl_2_]­BF_4_ on the metabolic activity of cell line **EA.hy926**.

**Figure 6:**
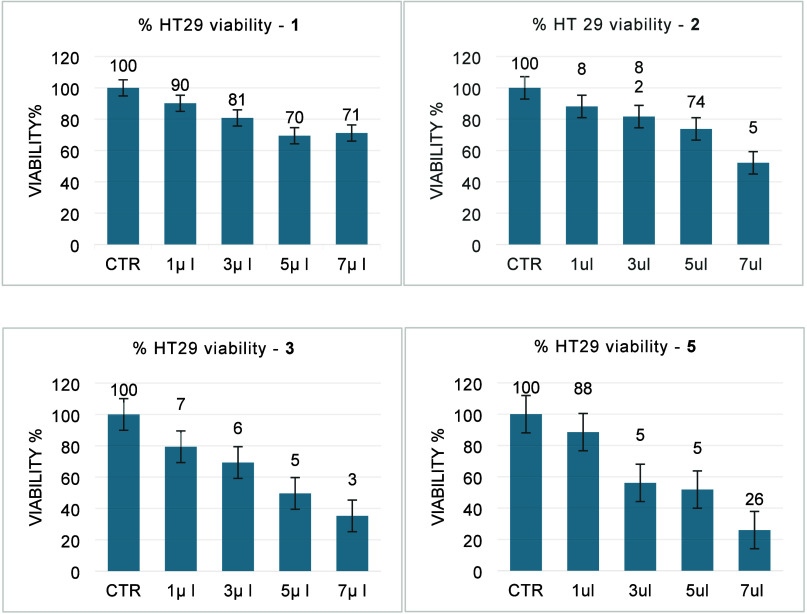
Effect of **1** [Pd­(**5-TzPy**)­Cl_2_], **2** [Pt­(**5-TzPy**)­Cl_2_], **3** [Pt­(**5-TzPy**)­Me_2_] and **5** Au­(**5-TzPy**)­Cl_2_]­BF_4_] on the metabolic activity of cancer cell line HT29.

All complexes tested cause a decrease in cell viability in a dose-dependent manner. Specifically, an increasing volume of the substance is inversely related to a progressive decrease in cell viability. Interestingly, for compounds **3** and **5** cell viability at our maximum test concentration (7 × 10^–4^ M) is decreased far below 40% (35% and 26% respectively) indicating the potential antitumor activity of these molecules. For complex **1** the results shown by the graph suggest a possible phenomenon of cell tolerance.

The human endothelial cells EA.hy926 cultured in Eagle’s minimum essential medium (EMEM) were used to evaluate cytotoxicity of the synthetised complexes. The molecules were tested at a final concentration of 0.01 M and evaluated by volume dilutions of 1 μL, 3 μL, 5 μL, and 7 μL. The results obtained in the EA.hy926 cells are reported in Figure 5. Treatment of EA.hy926 with palladium complex **1** induces an evident decrease in cell viability (from 79% to 27%), unlike that observed in the HT29 cells, where only a slight cell viability reduction was observed (from 90% to 71%), supporting a possible mechanism of tolerance. Noteworthy, for complex **2**, no reduction in % of viability was observed in the different samples, indicating a very low toxicity of the molecule, at least for the EA.hy926 cells. This trend is different from that obtained in the HT29 cells, where an evident reduction in cell viability in a dose-dependent manner was observed (from 88% to 52%). For compound **3**, whose behavior in solution is dynamic and has been noted above, the data show a clear decrease in cell viability in a dose-dependent correlation (from 81% to 17%), similarly to what was obtained with the HT29 cells (from 79% to 35%) indicating a level of cytotoxicity for both types of cells. Finally, the treatment with complex **5** results in decreasing cell viability (from 78% to 32%) similar to that obtained in the HT29 cells (from 88% to 26%) indicating a nonspecificity between the two types of cells.

## Conclusions

Over the past few decades, the escalating antimicrobial resistance (AMR) crisis has driven an intensive search for alternative therapeutic compounds, with noble metal complexes emerging as a premier class of candidates. In this study, we synthesized and characterized a series of platinum­(II), palladium­(II), and gold­(III) complexes chelated with the ligand 2-(1-benzyl-1*H*-1,2,3-triazol-4-yl)­pyridine, **5-TzPy**. A key highlight of this work is the optimized synthetic strategy: by employing a one-pot, two-step reaction, we isolated pure products through a simplified workup. This approach significantly reduces costs and time by avoiding demanding purification steps, aligning the protocol with the principles of efficient and sustainable chemistry. Biological screening across antibacterial, antifungal, and cytotoxic assays yielded promising results. The cationic gold­(III) complex **5** exhibited the highest efficacy, characterized by the lowest MIC and MBC values. Notably, its superior ability to inhibit biofilm formation suggests that its ionic charge and specific chemical configuration significantly enhance the molecular diffusion and interaction with microbial targets. Electrochemical data provided a clear distinction between the species; while the palladium and platinum complexes (**1** and **2**) remained stable, complex **5** was easily reduced. This distinct redox behavior points toward an oxidative-stress-based mechanism (MoA), offering a robust chemical explanation for its potent bioactivity. Cytotoxicity studies on normal (EA.hy926) and tumor (HT29) cells revealed a strategic versatility within the series. Platinum­(II) complex **2** demonstrated low general toxicity paired with significant activity against cancer cells, while complexes **3** and **5** showed high tumor cell toxicity. The antimicrobial activity of the Pt­(II) precursor [Pt­(DMSO)_2_Me_2_] has been also tested, showing promising perspectives of investigation.

In conclusion, these findings underscore the potential of such N^∧^N noble metal complexes not only as powerful tools against resistant microbes and biofilms but also as selective and safer antitumor agents. The structural modularity of this scaffold provides a promising foundation for the future development of next-generation metalladrugs with tailored pharmacological profiles.

## Experimental Section

Unless otherwise stated, all reagents were purchased from commercial sources and used without further purification. The ^1^H and ^13^C NMR spectra were recorded with either a 600 MHz Bruker xx or 400 MHz Bruker Avance III 400 spectrometer. 2D COSY, NOESY, HSQC and HMBC NMR spectra were registered using standard pulse sequences. Chemical shifts were reported in ppm and referenced to TMS. Coupling constants are reported in Hz. Registration of the NMR spectra was carried out by Maria Orecchioni (Department of Medicine, Surgery and Pharmacy, UNISS). Conductivity measurements were carried out with a Philips PW 9505 conductimeter at 298 K.

Cyclic voltammetry experiments were carried out in a three-electrode, single compartment cell with an AUTOLAB PGSTAT12 instrument using the specific software NOVA 2.1. The working electrode was a Pt disk (diameter 2 mm); a graphite bar was the counter electrode, and Ag/AgCl with a suitable salt bridge was the reference electrode. Before each experiment, the working electrode was polished with 1 and 0.3 μm alumina powder, then rinsed with distilled water in an ultrasonic bath, and finally rinsed with acetone. The concentration of the complex was 1.5 × 10^–3^ M in CH_2_Cl_2_ (anhydrous, packaged under nitrogen) as solvent and 0.1 M tetraethylammonium hexafluorophosphate (TEAPF_6_) as supporting electrolyte. All of the electrochemical tests were performed at room temperature under an argon atmosphere. All potential values are referred to the half-wave potential of the ferrocene/ferricinium ion (Fc/Fc^+^) redox couple (E_1/2_ = 0.485 V vs Ag/AgCl).

### Syntheses

Synthesis of 2-(1-benzyl-1*H*-1,2,3-triazol-4-yl)­pyridine (**5-TzPy**): A mixture of benzyl-bromide (3.5 mmol, 0.6 g) and sodium azide (5.2 mmol, 0.34 g) in 30 mL of a ^*t*^ButOH/H_2_O (1/1) mixture is heated at 50 °C for 5 h. Stoichiometric AgNO_3_ (3.5 mmol, 0.6 g) is added to the mixture, and the resulting precipitated AgBr is filtered off. The obtained solution is directly used for the CuAAC reaction. Therefore, sodium ascorbate (3.2 mmol, 0.6 g) has been added to the ^*t*^ButOH/H_2_O (1/1) mixture of (azidomethyl)­benzene under an inert atmosphere. After addition of ethynyl-pyridine (3.2 mmol, 0.33 g) and Cu­(OAc)_2_·H_2_O (0.32 mmol, 0.064 g), the reaction mixture was stirred at 60 °C for 12 h. At the end of the reaction the solvent has been removed under vacuum. The residual has been dissolved with DCM, and the organic phase, washed with 1 N aqueous EDTA to remove the copper salt. Finally, the organic phase has been dried with MgSO_4_ and filtered, and the solvent removed under vacuum to yield pure 2-(1-benzyl-1*H*-1,2,3-triazol-4-yl)­pyridine **5-TzPy** as a pale brown solid (88% isolated yield). ^1^H NMR (600 MHz, CDCl_3_) δ 8.53 (d, J = 4.9 Hz, 1H, H^6^), 8.19 (d, J = 10.2 Hz, 1H, H^3^), 8.12 (s, 1H, H^5′^), 7.77 (td, *J* = 7.7, 1.8 Hz, 1H, H^4^), 7.23–7.20 (m, 5H, H^2”‑6”^), 7.22 (m, partially overlapping, 1H, H^5^), 5.57 (s, 2H, C*H*_*2*_). ^13^C NMR (151 MHz, CDCl_3_) δ: 150.1, 149.1, 148.5, 137.4, 134.4, 129.3 (2C), 128.9, 128.4 (2C), 123.0, 122.3, 120.5, 54.5. MS (EI): *m*/*z* 236.1 [M]^+^.

#### Synthesis of [Pd­(**5-TzPy**)­Cl_2_], **1**

To a solution of [Pd­(COD)­Cl_2_] (73.8 mg, 0.26 mmol) in CH_2_Cl_2_ (20 mL) 68.6 mg of **5-TzPy** (0.26 mmol) were added under an argon atmosphere. The mixture was stirred at room temperature for 1 h; after that the precipitate formed was filtered under vacuum and washed with diethyl ether. Yield 80%. ^1^H NMR (DMSO-d_6_): δ 9.21 (s, 1H, H^5′^); 8.97 (d, 1H, H^6^, J = 5.8 Hz); 8.29 (dt, 1H, J = 7.9 Hz, J = 1.5 Hz); 8.20 (d, 1H, J = 7.9 Hz); 7.72–7.67 (m, 1H); 7.49–7.38 (m, 1H); 5.86 (s, 2H, CH_2_).

#### Synthesis of [Pt­(**5-TzPy**)­Cl_2_], **2**

To a solution of [Pt­(DMSO)_2_Cl_2_] (142.1 mg, 0.34 mmol) in acetone (25 mL) 80.1 mg of **5-TzPy** (0.34 mmol) were added under vigorous stirring. The mixture was stirred under an inert atmosphere for 5 h, then concentrated to small volume, and treated with diethyl ether. The precipitate formed was filtered under a vacuum and washed with diethyl ether to give the analytical sample as a yellow solid. Yield 70%. ^1^H NMR (DMSO-d_6_) δ 9.34 (d, 1H, H^6^, J = 5.7 Hz); 9.24 (s, 1H, H^5′^); 8.34 (td, 1H, H, J = 7.7 Hz, J = 1.3 Hz); 8.23 (d, 1H, H, J = 7.8 Hz); 7.75–7.69 (m, 1H); 7.52–7.40 (m, 6H); 5.85 (s, 2H, HCH_2_).

#### Synthesis of [Pt**(5-TzPy**)­Me_2_], **3**

A solution of [Pt­(DMSO)_2_Me_2_] (160.3 mg, 0.42 mmol) and **5-TzPy** (102.2 mg, 0.43 mmol) in acetone (20 mL) was stirred at room temperature for 1 h under an argon atmosphere. After that the solution was concentrated to a small volume and treated with diethyl ether. The precipitate formed was filtered under vacuum and washed with diethyl ether to give the analytical sample as a yellow solid. Yield 75%. Melting point: 222 °C. NMR characterization based on ^1^H, H–H COSY, H–H NOESY, ^13^C, H–C HSQC and H–C HMBC experiments. Elemental analysis: expected C 41.65%, H 3.93%, N 12.14%; found, C 41.33%, H 3.81%, N 11.87%. ^1^H NMR (CDCl_3_) δ 9.12 (d, 1H, H^6^, J = 5.4 Hz); 7.97 (td, 1H, H^4^, J = 7.7 Hz, J = 1.6 Hz); 7.81 (s, 1H, H^5′^); 7.55 (dt, 1H, H^3^, J = 7.8 Hz, J = 0.9 Hz); 7.48–7.31 (m, 6H, H^Ph^ and H^5^); 5.60 (s, 2H, CH_2_); 1.34 (s, 3H, H^Me′′^); 1.05 (s, 3H, H^Me’^). ^13^C NMR (CDCl_3_) δ 149.36 (Cq), 147.22 (c^6^), 136.49, 132.87, 129.63, 129.49, 128.47, 125.38, 121.79, 120.63, 55.64 (CH_2_), −17.67 (Pt-CH_3_), −24.30 (Pt-CH_3_). 2D NMR correlations: ^13^C–^1^H HSQC (CDCl_3_) selected data: δ 147.22 (C^6^, correlated to ^1^H NMR signal at 9.12 ppm); 55.64 (CH_2_, correlated to ^1^H NMR signal at 5.60 ppm); −17.41 (Me′′, correlated to ^1^H NMR methyl signal at 1.05 ppm); −24.59 (Me′, correlated to ^1^H NMR methyl signal at 1.34 ppm.

#### Synthesis of [Au­(**5-TzPy**)­Cl_3_], **4**

A mixture of Na­[AuCl_4_] (100.4 mg, 0.25 mmol) and **5-TzPy** (61.6 mg, 0.26 mmol) in 15 mL of a mixture H_2_O/CH_3_CN 3:1 was stirred at room temperature in the dark for 16 h. The precipitate formed was filtered under a vacuum and dried to give the analytical sample as a yellow solid. Yield *ca* 85% with a 5% of [Au­(**5-TzPy**)­Cl_2_]­Cl. ^1^H NMR (acetone-d_6_): δ 9.22 (s, 1H, H^6^, J = 7.9); 9.06 (s, 1H, H^5′^); 8.41–8.31 (m, 2H); 7.88 (t, 1H); 7.56–7.36 (m, 5H); 5.88 (s, 2H, CH_2_).

#### Synthesis of [Au­(**5-TzPy**)­Cl_2_]­BF_4_, **5**

To a solution of [Au­(**5-TzPy**)­Cl_3_] (89.2 mg, 0.16 mmol) in acetone (80 mL) was added a solution of AgBF_4_ (30.2 mg, 0.16 mmol) in acetone (10 mL) under vigorous stirring. The AgCl formed was filtered off, and the solution evaporated to dryness. The solid residue was crystallized from CH_2_Cl_2_/Et_2_O to give the analytical sample as a yellow solid. Yield 85%. M.P. = 135–137 °C. Elemental analysis: expected C 28.45%, H 2.05%, N 9.48%; found, C 28.26%, H 2.24%, N 9.19%. ^1^H NMR (acetone-d_6_): δ 9.57 (s, 1H, H^5′^); 9.51 (s, 1H, H^6^, J = 5.5 Hz); 8.84–8.70 (m, 2H); 8.22 (t, 1H); 7.69–7.61 (m, 2H); 7.56–7.48 (m, 3H); 6.15 (s, 2H, H^CH2^). ^13^C NMR (acetone-d_6_): δ 150.16 (Cq); 149.32 (C^6^); 147.73; 134.40; 131.40; 131.06 (C^Ph^); 130.81 (C^Ph)^; 129.98; 129.54; 126.80; 59.23 (CH_2_). Λ_M_ (acetone, 5 × 10^–4^ M) = 123 ohm^–1^ cm^2^ mol^–1^_._

### Antimicrobial Assay

The antimicrobial activity of compounds solubilized in 1 mL dimethyl sulfoxide (concentration = 0.01 M) was tested against Gram-positive (*Streptococcus pyogenes* DSM 20565, *Staphylococcus aureus* DSM 1104), Gram-negative (*Klebsiella pneumoniae* DSM 681, *Escherichia coli* DSM 1103 and *Pseudomonas aeruginosa* DSM 1117) and yeast (*Candida albicans* DSM 1386) strains, revealing promising antibacterial and antibiofilm properties. 50 μL of the frozen bacterial suspension were inoculated in Petri dishes containing Mueller Hinton Agar (aerobic strains) and Sabouraud Dextrose Agar (*C. albicans*) and incubated in air at 37 °C for 24 h, while the microaerophilic species were sown in Petri dishes containing Shaedler Agar (*S. pyogenes*) and incubated in 5% CO_2_ at 37 °C. A preliminary assessment of antimicrobial activity was carried out using the agar diffusion method (Kirby–Bauer test). Each strain was inoculated onto the surface of the plate using a sterile buffer, with a standardized bacterial inoculum of 5 × 10^7^ CFU and 5 × 10^6^ CFU for yeast. Three wells were used for each tested molecule, and two for the negative control. Petri dishes were incubated in air at 37 °C for 24 h for aerobic strains and in 5% CO_2_ at 37 °C for microaerophilic species. After incubation, we measured the diameters of the inhibition halo,^22^ as they are proportional to the logarithm of each molecule concentration [mol/L].

### Minimum Inhibitory Concentration (MIC) and Minimum Bactericidal Concentration (MBC) Tests

This evaluation was performed in sterile 96-well microplates, with each well containing serial dilutions (0.5% to 0.0004%) of each compound dissolved in nutrient broth. Briefly, serial concentrations of 0.01 M of each compound were tested, and the final concentration of the strains was 1 × 10^6^ CFU/mL. The experiment was repeated three times. After 24–48 h of incubation at 37 °C in an appropriate atmosphere (air or 5% CO_2_), the minimum inhibitory concentration (MIC) was defined as the lowest concentration of the tested compound that inhibited visible growth; i.e., the well showed the same absorbance (550 nm) as the negative control, measured with a Multiskan FC microplate photometer (Thermo Fisher Scientific IT, Milan, Italy). After determining the MIC of compounds, aliquots of 50 μL from all wells that showed no visible bacterial growth were seeded onto Mueller-Hinton agar plates (for *E. coli*, *K. pneumoniae*, *P. aeruginosa* and *S. aureus*), Shaedler agar plates (for *S. pyogenes*) and Sabouraud (for *C. albicans*). The agar plate was incubated for 24 h at 37 °C.

### Antibiofilm Assay

Minimum inhibitory concentration of biofilm (MBIC) was evaluated following the crystal violet staining protocol described by the Montana University Center for Biofilm Engineering (http://www.biofilm.montana.edu) with some modifications. Therefore, after 48 h of incubation in an appropriate atmosphere (air or 5% CO_2_), the medium was discarded and the wells were gently washed three times with a 0.9% NaCl solution. Then 0.1 mL of a 0.1% crystal violet solution was added to each well; after 10 min the dye was discarded, followed by three washes with 0.9% NaCl and solubilized with 200 μL acetic acid (30%). Finally, the absorbance of the biofilm was measured at 620 nm with a Multiskan FC microplate photometer (Thermo Fisher Scientific IT, Milan, Italy).

### Cytotoxicity Assay

The human endothelial cells EA.hy926 were cultured in Eagle’s minimum essential medium (EMEM), supplemented with 10% vol/vol FBS, 100 units/ml penicillin, and 100 μg/mL streptomycin was used as a controls value. The cells were incubated at 37 °C in 95% humidified air with 5% CO_2_ for 2–3 days. Cells were seeded in 96-well plates at a density of 1.5 × 10^–4^ cells per well in 100 μL of complete medium. The EA.hy926 cells were treated with the same solution of the different substances used in the H29 cancer cells. The complexes were tested at a final concentration of 0.01 M and evaluated by volume dilutions of: 1 μL, 3 μL, 5 μL, and 7 μL. Confluent HT29 cells were isolated using trypsin/EDTA, and 1.5 × 10^–4^ cells/cm^2^ were plated with a mixture of RPMI 1640 10% fetal bovine serum (FBS), 100 units/mL penicillin, 100 μg/mL streptomycin, and 2 mM l-Glutamine ND 1% nonessential amino acids. Commercial human cell line HT29 (ATCC-HTPS: //wwwatcc.org) was obtained from the Istituto Nazionale per la Ricerca sul Cancro c/o CBA (ICLC, Genova). Different amounts of metal compound were added, and after 24 h the MTT viability test was performed using the cell proliferation Kit I (MTT), Roche REF number 11465007001. Different complexes were dissolved at 0.01 M final concentration, and 1 μL, 3 μL, 5 μL, and 7 μL of these solutions were added to the in vitro medium used. The viability control value obtained in untreated cells was considered as 100% of viability.

## Supplementary Material



## Notes

Figure S1: ^1^H NMR spectrum (CDCl_3_) of 5-TzPy; Figure S2: ^13^C NMR spectrum (CDCl_3_) of 5-TzPy; Figure S3: ^1^H NMR spectrum (DMSO-d6) of **1**, [Pd­(5-Tzpy)­Cl_2_]; Figure S4: ^1^H NMR spectrum (DMSO-d6) of **2**,[Pt­(5-Tzpy)­Cl_2_]; Figure S5: ^1^H NMR spectrum (CDCl_3_-d6) of **3,** [Pt­(5-Tzpy)­Me_2_]; Figure S6: ^1^H COSY NMR spectrum (CDCl_3_) of **3**, [Pt­(5-Tzpy)­Me_2_; Figure S7: ^1^H NMR NOESY spectrum (CDCl_3_) of **3**, [Pt­(5-Tzpy)­Me_2_]; Figure S8: ^1^H–^13^C HSQC spectrum (CDCl_3_) of **3**, [Pt­(5-Tzpy)­Me_2_]; Figure S9: ^1^H NMR spectrum (acetone-d6) of **5**, [Au­(5-Tzpy)­Cl_2_]­BF_44_. (PDF)
