# Supplementary material for: Synthesis, Characterization, and Biological Evaluation of Noble Metal Complexes with 2‑(1-Benzyl‑1H‑1,2,3-triazol-4-yl)pyridine Ligand: An Interesting Class of Metallo-antimicrobial and -antitumor Agents
Source: ACS Omega. 2026 Apr 16;11(16):24597–610. doi: 10.1021/acsomega.6c00770 (PMC13129826; doi:10.1021/acsomega.6c00770)
Supplement: Supplementary file 1 [file ao6c00770_si_001.pdf]

Supporting information

Synthesis, characterization, and biological evaluation of noble metal complexes with 2-(1-benzyl-1*H*-1,2,3-triazol-4-yl)pyridine ligand: an interesting class of metallo-antimicrobial and -antitumor agents

Daniela Giunta[a], Maurizio Solinas[a], Bruna Canu[b], Maria I. Pilo,[b] Alessandra Scano[c], Germano Orrù,[c] Sara Fais,[c] Giuseppina Pichiri[d] and Antonio Zucca[b]

a) Institute of Biomolecular Chemistry, National Research Council (CNR), Trav. La Crucca 3, 07100, Sassari, Italy
b) Department of Chemical, Physical, Mathematical and Natural Sciences, University of Sassari, Via Vienna 2, 07100 Sassari, Italy
c) Department of Surgical Sciences, University of Cagliari, 09124 Cagliari, Italy
d) Department of Medical Sciences and Public Health, University of Cagliari, 09124, Cagliari, Italy.

NMR spectra

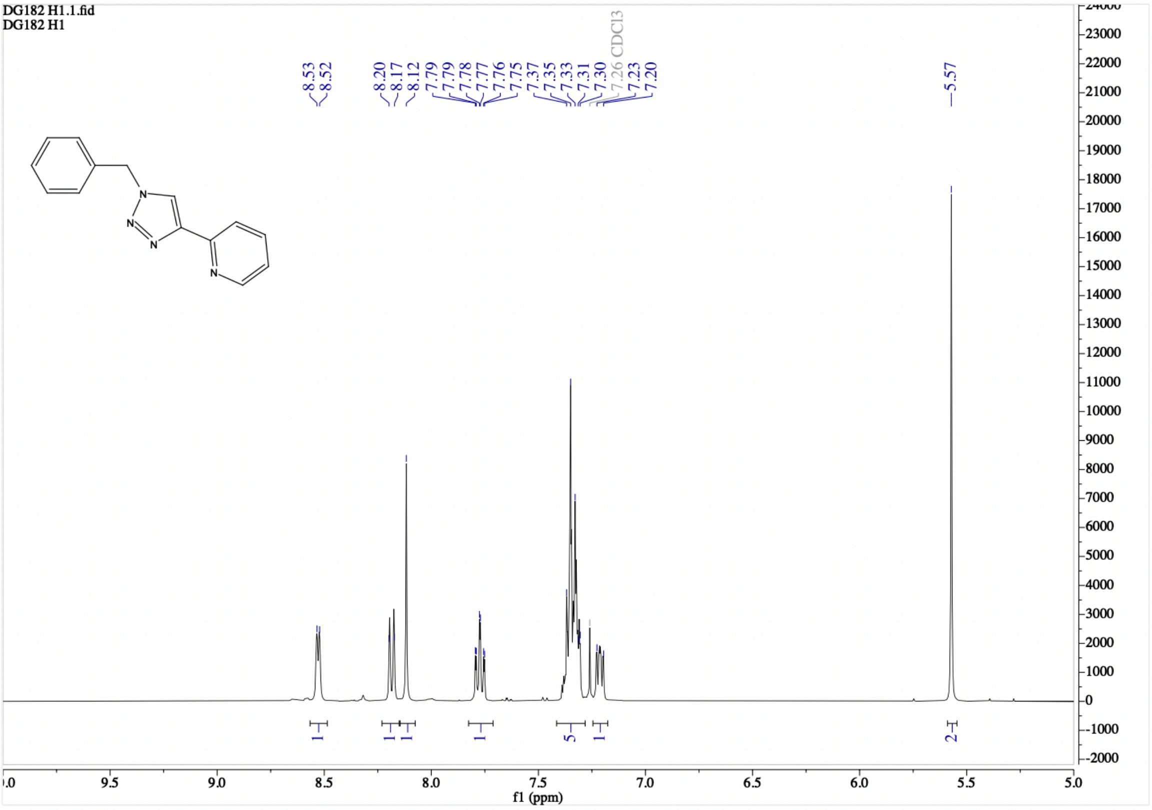

Figure S1. ^{1}H NMR spectrum ($CDCl_3$) of **5-TzPy**

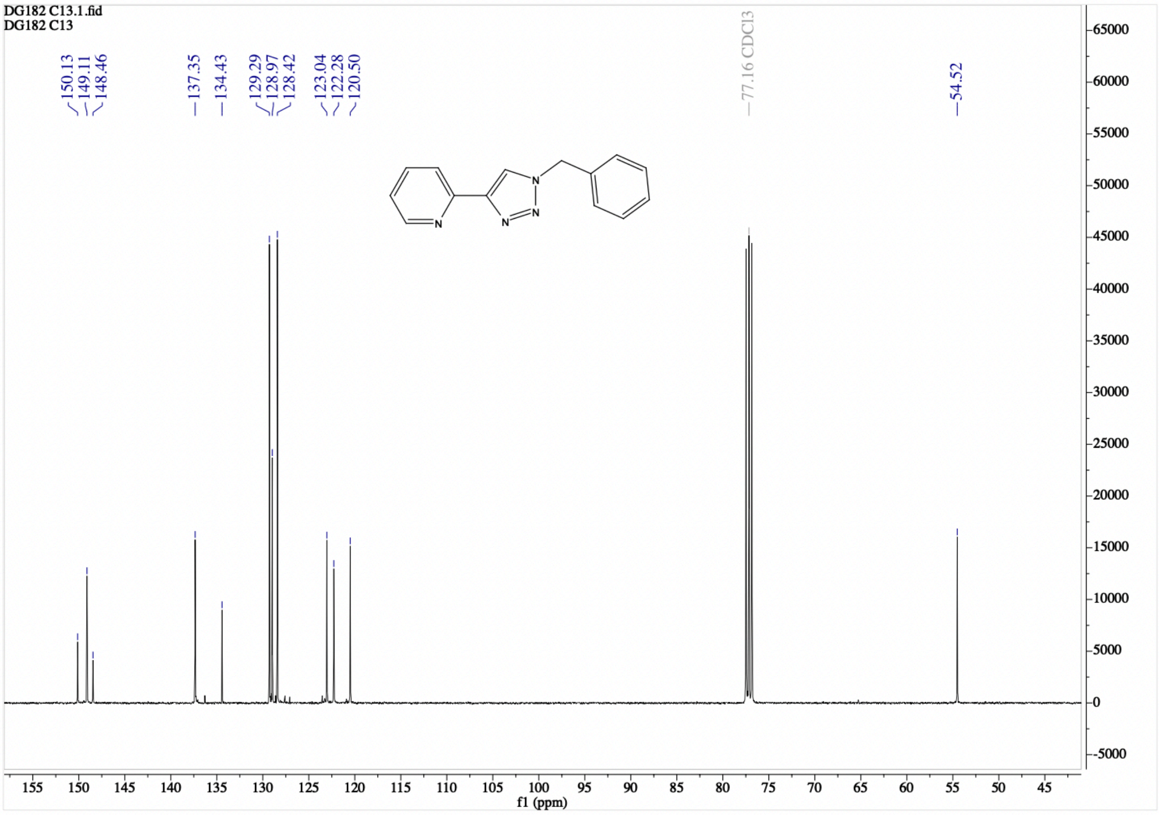

Figure S2. ^{13}C NMR spectrum ($CDCl_3$) of **5-TzPy**

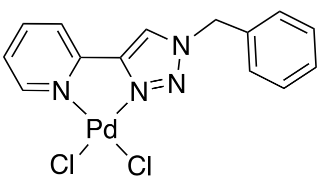

1

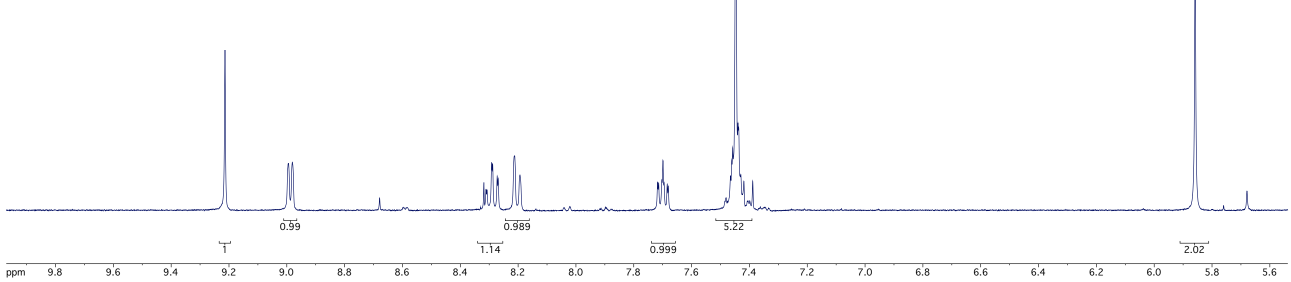

Figure S3. ^{1}H NMR spectrum (DMSO-d6) of **1** [**Pd(5-Tzpy)Cl_2**]

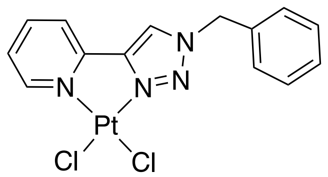

2

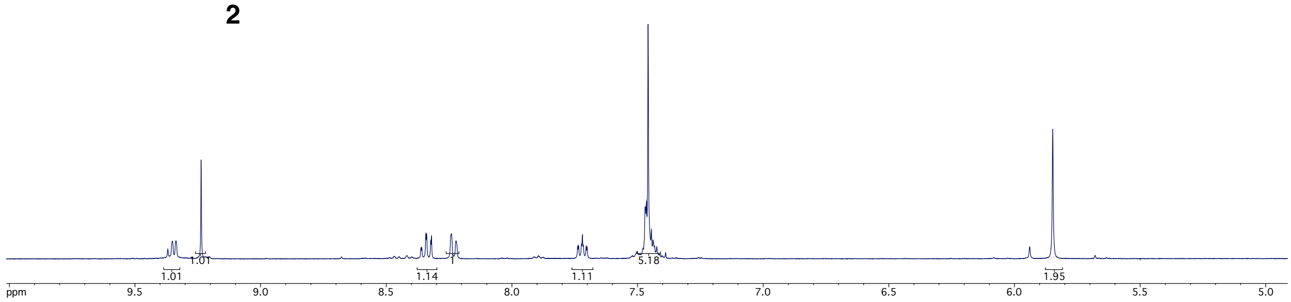

Figure S4. ^{1}H NMR spectrum (DMSO-d6) of **2** **[Pt(5-Tzpy)Cl_2]**

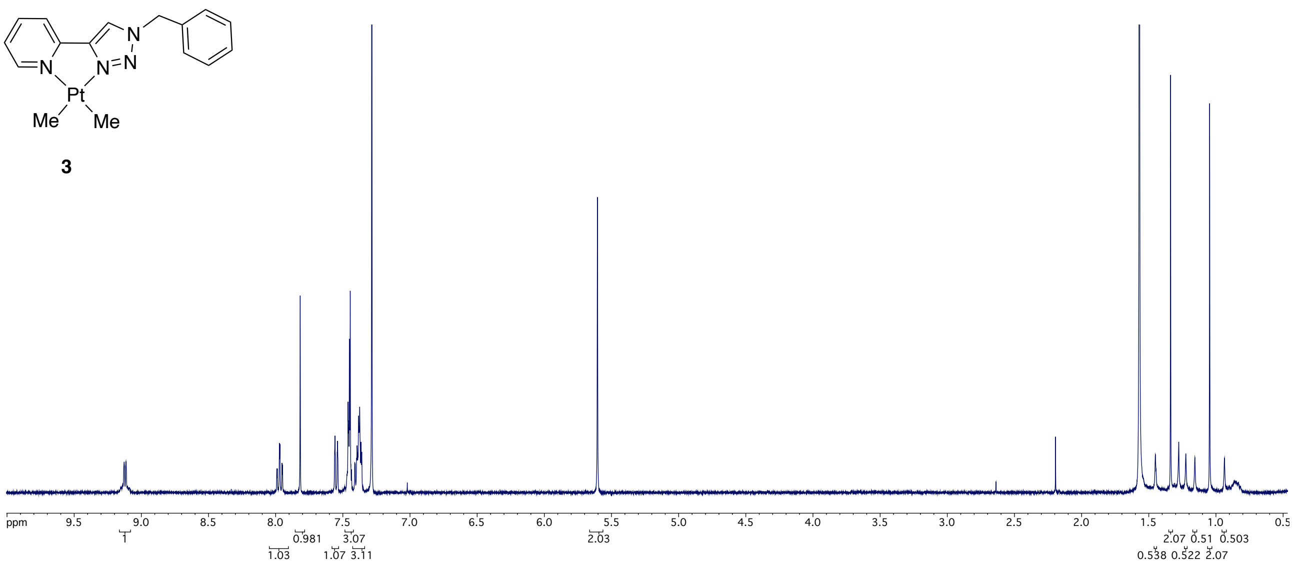

Figure S5. ^{1}H NMR spectrum ($CDCl_3$-d6) of **3** [**Pt(5-Tzpy)Me$_2$]**

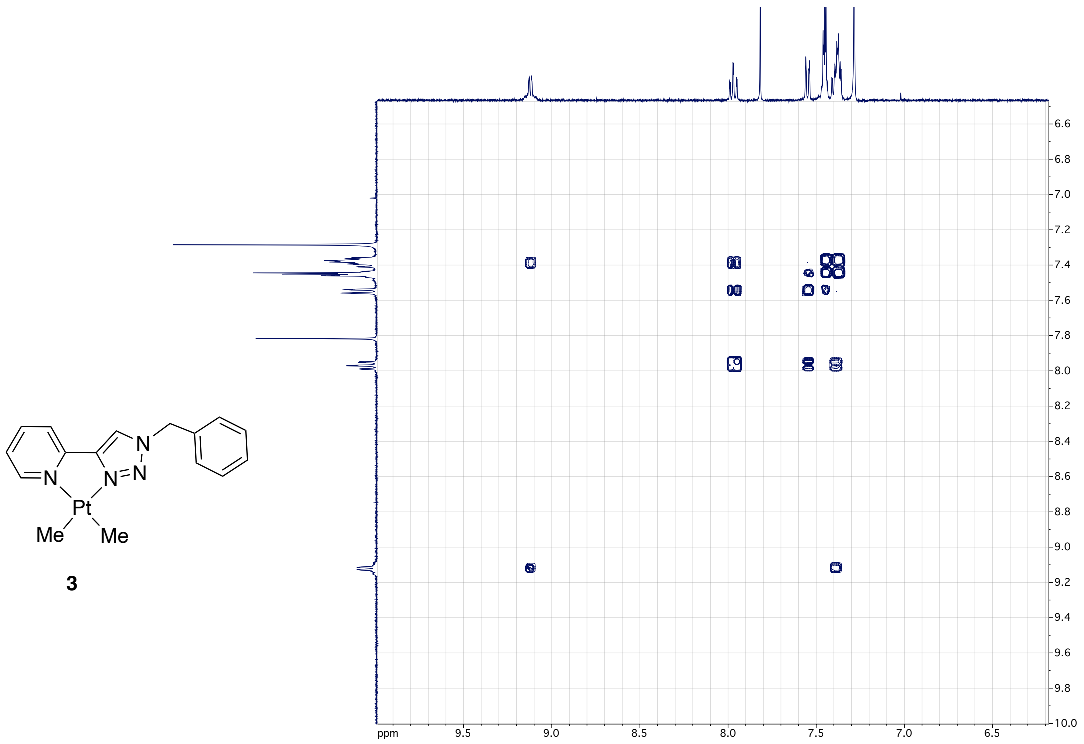

Figure S6. ^{1}H COSY NMR spectrum ($CDCl_3$) of **3** [**Pt(5-Tzpy)Me$_2$**

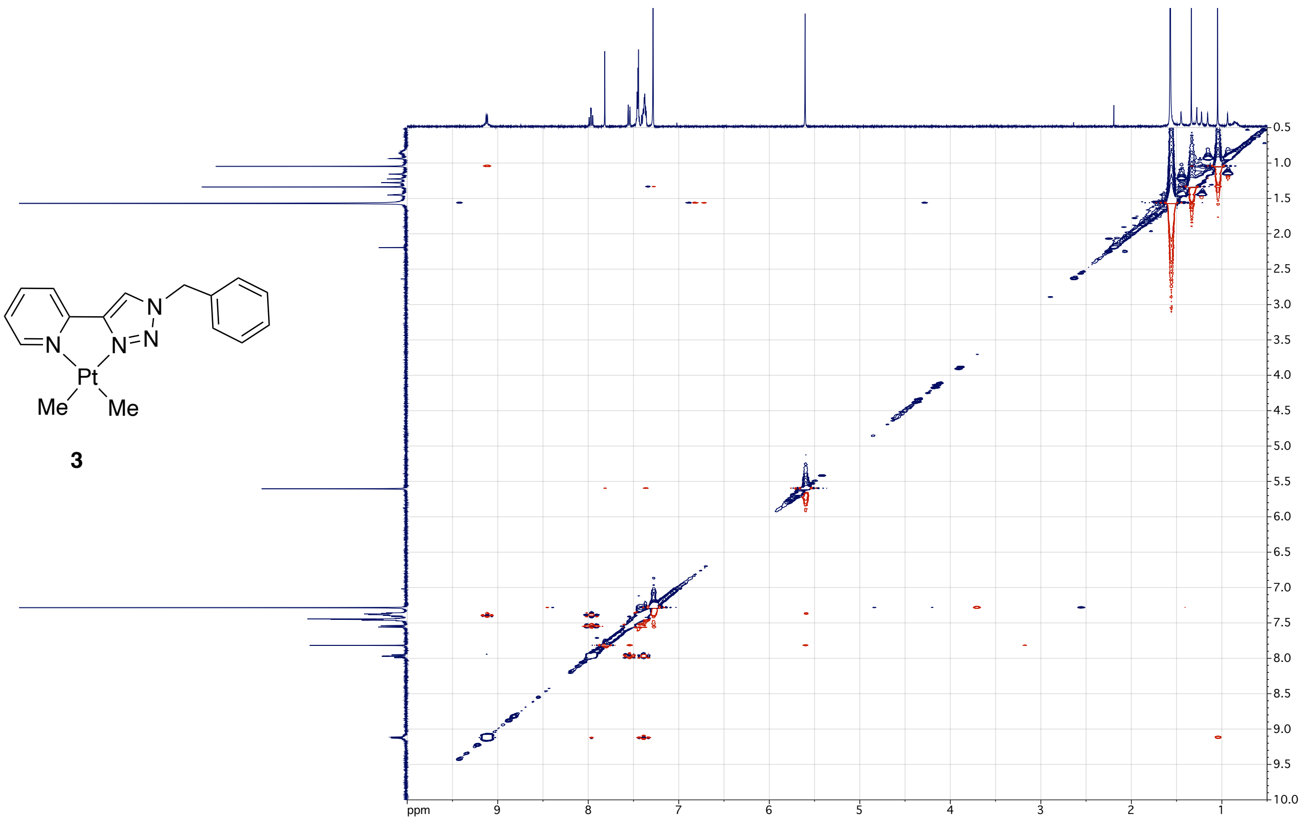

Figure S7. ^{1}H NMR NOESY spectrum ($CDCl_3$) of **3** [**Pt(5-Tzpy)Me$_2$**]

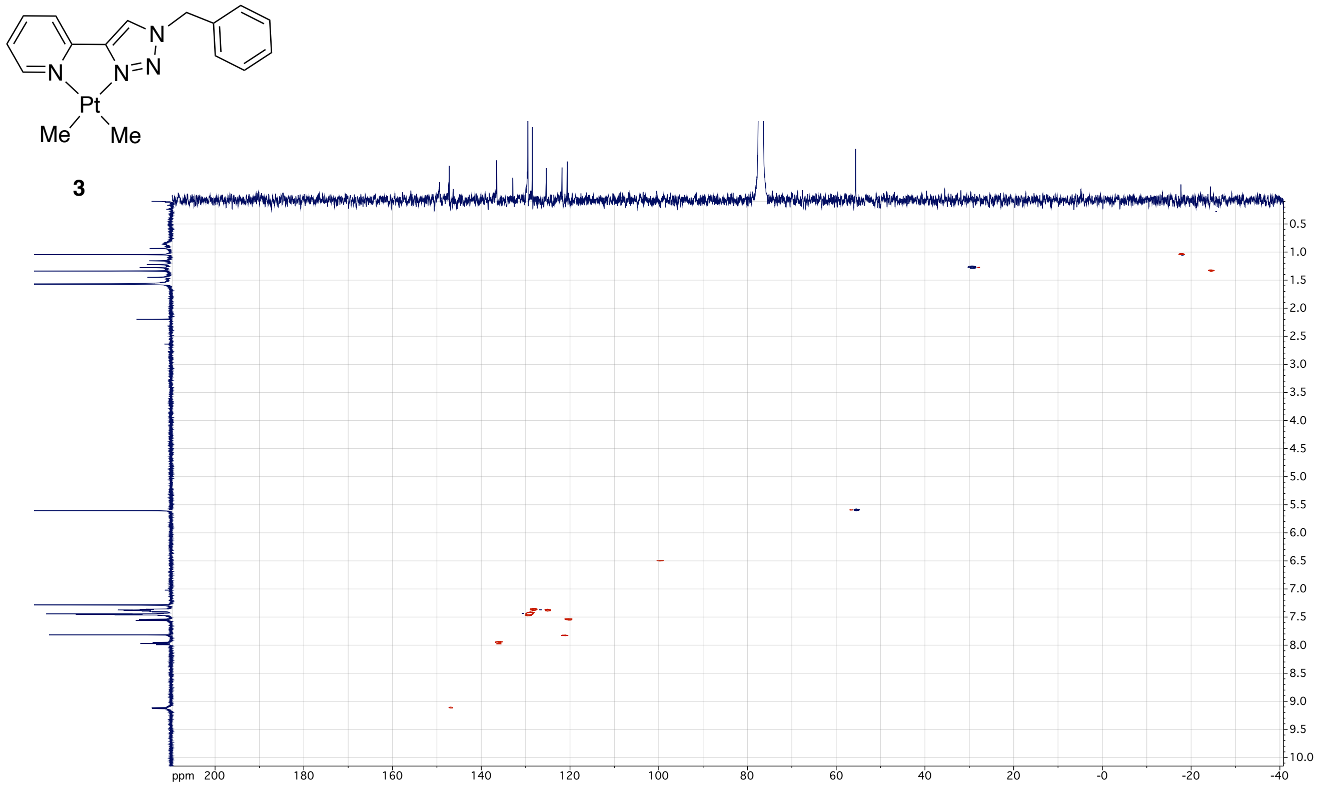

Figure S8. ^{1}H-^{13}C HSQC spectrum ($CDCl_3$) of **3** [**Pt(5-Tzpy)Me$_2$**]

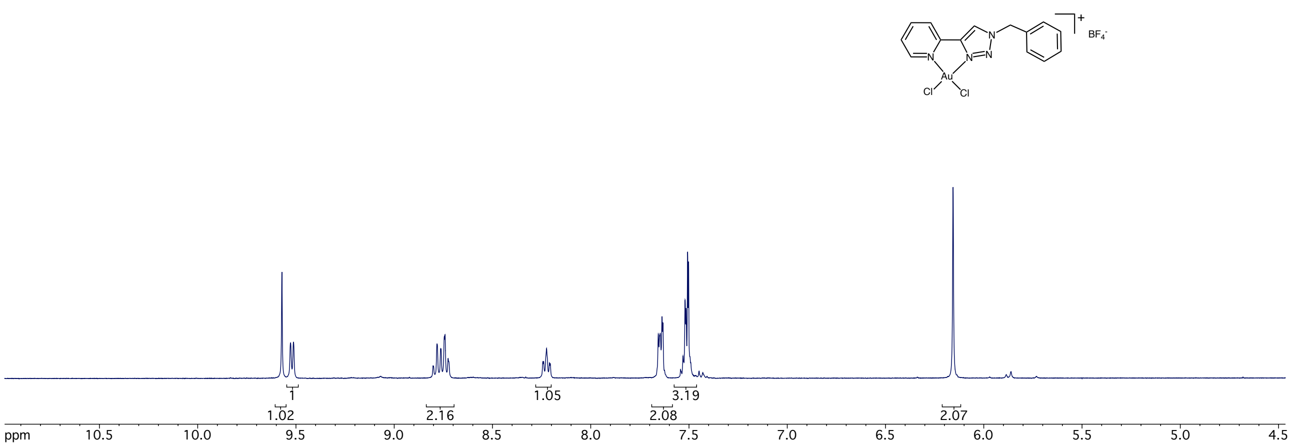

Figure S9. ^{1}H NMR spectrum (acetone-d6) of **5 [Au(5-Tzpy)Cl$_2$]BF$_4$**
